# Weather, air pollution, and migraine: A case-time series analysis examining environmental exposures and transient health outcomes recorded via smartphone application

**DOI:** 10.1097/EE9.0000000000000475

**Published:** 2026-05-08

**Authors:** Andrea E. Portt, Antonio Gasparrini, Erjia Ge, Christine Lay, Hong Chen, Peter M. Smith

**Affiliations:** a Dalla Lana School of Public Health, University of Toronto, Toronto, Ontario, Canada; b Department of Public Health, Environments and Society, London School of Hygiene and Tropical Medicine, London, United Kingdom; c Centre for Headache, Women’s College Hospital, Toronto, Ontario, Canada; d Neurology, Temerty Faculty of Medicine, University of Toronto, Toronto, Ontario, Canada; e Environmental Health Science and Research Bureau, Health Canada, Ottawa, Ontario, Canada; f Public Health Ontario, Environmental and Occupational Health, Toronto, Ontario, Canada; g Populations and Public Health Research Program, ICES, Toronto, Ontario, Canada; h Institute for Work and Health, Toronto, Ontario, Canada

**Keywords:** Air pollution, Weather, Migraine, Smartphone application data, Mobile data, Short-term exposure, Case-time series

## Abstract

**Background:**

Most studies of environmental exposures and migraine have been limited to weather or single-pollutant models and aggregate outcome measures. We estimated the associations between multiple environmental exposures and migraine events captured using a smartphone application (app) in the province of Ontario, Canada.

**Methods:**

We obtained Ontario-wide local daily weather and pollution estimates for 2017–2019 from Environment and Climate Change Canada. The Migraine Buddy app team provided records from research-consenting users. Associations between environmental exposures and migraine attack onsets were examined with case-time series lagged multipollutant models, accounting for demographic and temporal covariates.

**Results:**

We analyzed 69,808 migraine attacks reported by 7418 participants in Ontario, Canada. Overall cumulative estimates for 0–3 days suggested increased odds for migraine attacks with higher nitrogen dioxide (NO_2_) and ozone (O_3_), drops in barometric pressure, and cold winter temperatures. No association was observed for PM_2.5_. Positive associations with NO_2_ and O_3_ were strongest 1 and 2 days after exposures, while associations with barometric pressure changes and cold winter temperatures were strongest on the first day. Estimates for warmer summer temperatures suggested a complex temporal pattern, with positive same-day association followed by 3 days of negative associations.

**Conclusion:**

This study presents a novel examination of the associations between air pollution and migraine attacks using small-area exposures, smartphone data, and the case-time series method. Onset of migraine was associated with higher NO_2_ and O_3_, colder winter weather, and positive and negative changes in barometric pressure.

**What this study adds**

The association between environmental exposures and migraine is inconclusive despite reviews.^1–4^ Studies of weather and migraine have yielded inconsistent results, with some reporting associations with temperature or barometric pressure^5^ and others disagreeing.^2^ For air pollution, previous work showed that point estimates were mainly positive for associations with carbon monoxide, nitrogen dioxide, ozone, particulate matter, and sulfur dioxide with migraine, however, with substantial uncertainty.^1,4^ Results of this study suggest that elevated exposures to nitrogen dioxide and ozone were associated with migraine attack onset at 1-day and 2-day lags. In our study, we also observed low winter temperatures and high summer temperatures, and large positive or negative changes in barometric pressure were associated with migraine attack onset on the same day (0-day lags). To our knowledge, previous work has not explored these questions using smartphone app data.

## INTRODUCTION

### Background

Migraine is a brain disease with episodic attacks of debilitating combinations of headache, nausea or vomiting, and sensitivity to light or sound.^6^ People living with migraine try to avoid or manage established triggers such as lack of sleep, stress, hormonal changes, and alcohol consumption.^7,8^ Knowledge of potential weather and air pollution triggers could allow people with migraine to manage these exposures and limit related attacks.

Questions remain about the biological pathways between environmental exposures and migraine attacks.^1,2^ For example, studies in rats have reported that changes in atmospheric pressure and temperature are linked to peripheral vasoconstriction, local increases in analgesic substances and adrenaline, and lower pain thresholds.^9–11^ In addition, a 2017 study reported increased firing in a subset of neurons from rat trigeminal ganglia during relatively cold temperatures, implying that temperature change may increase excitability in pain-related trigeminal neurons.^12^ In contrast with these neuron-mediated pathways from weather to migraine, the pathophysiology connecting air pollution and migraine is thought to be mediated by oxidative stress.^7,13,14^ Two studies have found that rats developed migraine-like behaviors and pathology after exposure to nitrogen dioxide (NO_2_).^15,16^ The changes appeared to result from upregulation of calcitonin gene-related peptide (CGRP),^16^ which is an established target for migraine treatments.^8^

Studies in humans have mostly examined weather and/or individual pollutants,^1,2^ despite the fact that these environmental exposures can be correlated.^17^ Work has also been limited to aggregated analyses, which cannot account for individuals’ movement in space or time.^1^ Moreover, the most common outcome measure has been emergency room visits for migraine,^1^ which have been demonstrated to vary significantly in their timing with relation to the initial onset of the migraine attack.^18–20^ Emergency room (ER) records may also be subject to missingness not at random due to age- and gender-related factors,^20^ or due to variable access to treatment outside of an ER environment. This potential misclassification of exposure timing poses challenges for assessing the causal relation between environmental exposures and migraine outcomes,^20^ creating uncertainty in the reported migraine/environment associations.^1,2^

### Objectives

We leveraged new data sources and statistical methods to estimate associations between meteorological and air pollution exposures and migraine onset. In lieu of jurisdiction-wide, location-aggregated data, we employed individual-level exposure and smartphone app migraine record outcome data on 10 km2 grids across a large geographic area. We used case-time series modeling to estimate the effects of multiple exposures simultaneously while accounting for repeated events.

We hypothesized that acute exposures to NO_2_, ozone (O_3_), and particulate matter smaller than 2.5 micrometres in diameter (PM_2.5_) and cold winter weather, hot summer weather, and changes in barometric pressure would be associated with migraine attack onset, controlling for individual, day of week, holidays, month, and year.

## METHODS

### Sample population and ethics

This project was approved by the University of Toronto Health Sciences Research Ethics Board, Human Protocol Number 38331.

Migraine Buddy (Aptar Digital Health, Congers, NY) is a smartphone application (app) designed to help people living with migraine. The app is free to download and has over 4 million users worldwide. Migraine Buddy users record their attacks, potential triggers, symptom severity, and attack duration. Migraine Buddy produces standardized reports of attack-related frequency and disability to facilitate conversations with clinicians. The paid version of the app can also carry out personal regression analysis based on each user’s recorded triggers (including food, sleep, stress, and since 2021—local weather conditions) in a given month to produce a report of which triggers may be most important for the individual.

Migraine Buddy users have the option to consent to share their data for research, which can be withdrawn through the app at any time. Approximately 60% of users consent to research. For this study, the team at Migraine Buddy shared anonymized headache diary data from research-consenting users throughout Ontario from 2017 to 2019. To further preserve privacy, locations were pooled to ~10 km2 grids (0.1 degrees of latitude) with a minimum of six participants per location-day before transfer to the study team. Therefore, records with fewer than five other users in a given 10 km2 area were excluded. Consistent with the study design based on within-subject comparisons, we restricted our sample to participants who had recorded at least one attack labeled “migraine” in the app. There were no restrictions based on age or gender.

### Exposure estimates

Environment and Climate Change Canada supplied estimated temperature, specific humidity, barometric pressure, and pollution (NO_2_, O_3_, and PM_2.5_) data on a 10 km2 grid for 2017–2019. Estimates were point estimates at 12:00 UTC (07:00 EST). Meteorological data were generated using the well-established Regional Deterministic Prediction System.^21,22^ The 24-hour change in barometric pressure was estimated by subtracting the previous day’s pressure from the current day’s.

### Outcome assessment

We identified stand-alone migraine days and the first migraine day of multiday attacks in each participant’s Migraine Buddy diary. Given that prodrome-related symptoms may precede migraine attacks by several hours or up to 2 days,^23–25^ any two migraine events recorded with less than a 2-day break between them were analyzed as relating to a single migraine attack onset from the first day of the earlier attack. A recent survey of Migraine Buddy users in the United States of America found that 92% met the International Classification of Headache Disorders, 3rd edition (ICHD-3) criteria for migraine diagnosis.^26^

### Demographics and covariates

Migraine Buddy users may optionally share their age and gender. For age, we considered values less than five and over 100 as missing. Since the analytic method accounts for stable individual variables such as age and gender (see below), we included participants who were missing these variables in the analysis.

### Statistical analysis

We linked the 10 km2 locations where participants recorded their attacks to local weather and pollution exposure estimates using ArcGIS Pro version 3.0.3 (ESRI, Redlands, CA)^27^ and R version 4.4.2 (R Foundation for Statistical Computing, Vienna, Austria).^28^ Pearson correlation coefficients were calculated to estimate the correlation between exposures.

To analyze the linked data, we used the case-time series method. The case-time series method is a generalization of the case crossover method. It was designed to model multiple longitudinal profiles of health outcomes and time-varying predictors as individual time series.^29^ It has been applied in studies of heat and mortality or hospitalization,^30–32^ aircraft noise and sedative consumption,^33^ and pollen and asthma.^29^ This method can compare individuals to themselves on the same day of the week within the specific month. This intrinsically accounts for demographic and other potential confounders that are consistent within the comparison time frame, such as smoking status or gender, which can have high levels of missingness in smartphone app data. Comparing participants to themselves within specific month-years accounts for the potential transitory nature of app-based reporting. Estimating the population at risk for reporting a migraine is not easily done with app data. However, given that the case-time series is based on comparing individuals to themselves, it does not require a known denominator over time,^29^ unlike traditional time series.

We used conditional logit regression and assumed a binomial distribution for the binary outcome corresponding to daily migraine onset. Our fixed-effects case-time series model simultaneously included temperature, barometric pressure change, NO_2_, O_3_, and PM_2.5_, while accounting for individual, month, year, day of week, holidays, and seasonal trends, see Equation 1.

The fixed-effect model relies on variation within individuals, and hence absorbs unmeasured time-invariant confounding,^34^ which is likely to be present in smartphone data. We investigated the nonlinearity of exposure-response relation by comparing Akaike’s information criterion (AIC) among models with 2–4 equal knots. Where 2 equal knots resulted in a lower AIC than 3, we also tested for linear exposure-response relation (equivalent to 1 knot). We used the same multiexposure model for all effect estimates.

(Equation 1)


$$\begin{array}{cc} & log\left(\frac{p}{1-p}\right)={\vec{\beta }}_{stratum}X^{stratum}+\beta_{2}X^{temp}+\beta_{3}X^{pressure\;change\;\left(nonlinear\right)}\\ & +\beta_{4}X^{nitrogen\;dioxide}+\beta_{5}X^{ozone}+\beta_{6}X^{particulate\;matter\;2\mathrm{.5}}\\ & +\beta_{7}X^{seasonal\;trends}+\beta_{8}X^{day\;of\;week}+\beta_{9}X^{holiday} \end{array}$$

*p* = probability of migraine onset.

stratum = X is a categorical variable for individual-month-year strata.

Beta vectors = corresponding coefficient vector.

We hypothesized that hotter summer temperatures would have a different association with migraine onset than warmer winter temperatures. Therefore, we analyzed the seasons separately for temperature. We set the summer season as June–July–August, while winter was December–January–February.

Results were reported as the odds ratio (OR) with corresponding 95% confidence intervals (95 % CI) per 10 ppb NO_2_ and O_3_, per 10 μg/m^3^ PM_2.5_, per 10°C temperature increase, and per 1000 Pascal change in barometric pressure. A previous systematic review reported stronger associations between air pollution and migraine over 0–3 day lags compared with longer (e.g., 7-day) lags.^1^ Therefore, we examined 0–3 day lags to capture the full lagged associations for all pollutants. Results were presented as overall cumulative effects, which demonstrate the net associations accounting for the whole lag period, and the 4-day (0–3 day) specific lag-responses to the exposures of interest. Lags were tested as linear or nonlinear associations. All estimates included control for all the other coexposures.

All analyses were carried out using the packages gnm^35^ and dlnm^36^ in R version 4.4.2.^28^ We used RStudio version 2024.12.1 + 467^37^ (Boston, MA). The manuscript was typeset in Quarto version 1.6.42^38^ (Boston, MA). Analytic code is available at https://github.com/aportt/weather_pollution_migraine.

## RESULTS

**Table 1.** Gender, age, and mean number of migraine attacks of participants who recorded at least one new migraine attack in Ontario during the study period (2017–2019)

Variable	N = 7,418
Gender, n (%)	
Women	4,946 (66.7%)
Men	519 (7.0%)
Missing	1,953 (26.3%)
Age, mean (range)	34.3 (6.2, 80.0)
Missing, n (%)	4,977 (67.1%)
Total migraine attacks	69,808
Recorded migraine attacks per individual, mean (range)	9.4 (1, 151)
Migraine days, total (average per attack)	194,778 (2.8)

Table 1 presents demographic information on the study sample. Participants were located throughout Ontario, with most study locations within Southern Ontario. During our study period, there were 7418 research-consenting participants who recorded migraine attacks. This included 4946 (66.7%) women, 519 (7.0%) men, and 1953 (26.3%) participants missing gender data. The mean age was 34.3 years, range 6.2–80.0, with 67.1% missing data. The mean number of attacks per individual during the study period was 9.4, with a range from 1 to 151. There were 194,778 migraine days in the dataset, with an average attack length of 2.8 days.

**Table 2.** Estimated ranges of environmental exposures (temperature, specific humidity, 24-hour change in barometric pressure, NO_2_, O_3_, and PM_2.5_) at study locations across Ontario provided on daily 10 km^2^ grids by Environment and Climate Change Canada

Variable	Minimum	Median	Maximum	IQR
Temperature	−38.8°C	6.1°C	29.1°C	17.0°C
Specific humidity	0.000 kg/kg	0.005 kg/kg	0.021 kg/kg	0.006 kg/kg
Change in barometric pressure	−4617.8 Pa	−10.3 Pa	4704.1 Pa	942.3 Pa
NO_2_	0.0 ppb	5.2 ppb	60.4 ppb	7.5 ppb
O_3_	0.8 ppb	27.5 ppb	103.1 ppb	12.7 ppb
PM_2.5_	0.0 µg/m^3^	5.6 µg/m^3^	68.5 µg/m^3^	7.2 µg/m^3^

Table 2 presents the ranges of environmental exposures during the observation period. The interquartile range (IQR) was 17.0°C for temperature, 0.006 kg/kg for specific humidity, 942.3 Pa for 24-hour change in barometric pressure, 7.5 ppb for NO_2_, 12.7 ppb for O_3_, and 7.2 ppb for PM_2.5_.

**Table 3.** Pearson correlation coefficients (*r*) for estimated environmental exposures (temperature, humidity, 24-hour change in barometric pressure, NO_2_, O_3_, and PM_2.5_) among 7418 participants in Ontario from 2017 to 2019

Variable	Temperature	Humidity	Barometric pressure change	NO_2_	O_3_	PM_2.5_
Temperature	1.00	0.92	−0.06	−0.08	0.10	0.05
Humidity		1.00	−0.07	−0.08	0.17	0.09
Barometric pressure change			1.00	0.06	−0.08	0.01
NO_2_				1.00	−0.42	0.73
O_3_					1.00	−0.01

Table 3 presents correlations between environmental exposures. As expected due to shared emission sources and chemical relation,^17^ there was moderate correlation between NO_2_ and PM_2.5_ (*r* = 0.73), and NO_2_ and O_3_ (*r* = −0.42). Temperature and humidity were highly correlated in this dataset, *r* = 0.92. In addition to this high correlation, we found that including humidity increased the AIC (decreased parsimony of the model), and so our main analyses excluded humidity. As a post hoc analysis, we compared estimated associations with temperature from models with and without humidity. Including humidity had little effect on estimates (See Supplementary Content; https://links.lww.com/PRSGO/E873). These results suggest that humidity does not mediate a great deal of the estimated association between air pollution and relatively higher summer temperatures. The remaining correlation coefficients were close to the null value of 0.

### Estimated associations between environmental exposures and migraine onset

Linear relations were the most parsimonious (lowest AIC) for all variables except barometric pressure change, where a model with 3 equal knots was the most parsimonious. Therefore, we present results below as figures of linear associations except for barometric pressure change, which we present as a three-dimensional graph of the estimated OR over the range of pressure change and the time lags. Time lags were modeled linearly, as nonlinear lag models failed to converge. The same model was used for all figures, and controls for barometric pressure, NO_2_, O_3_, PM_2.5_, seasonal trends, month, year, day of week, and holidays.

### Four-day cumulative and specific-day lagged exposure-response estimates

**Figure 1.**
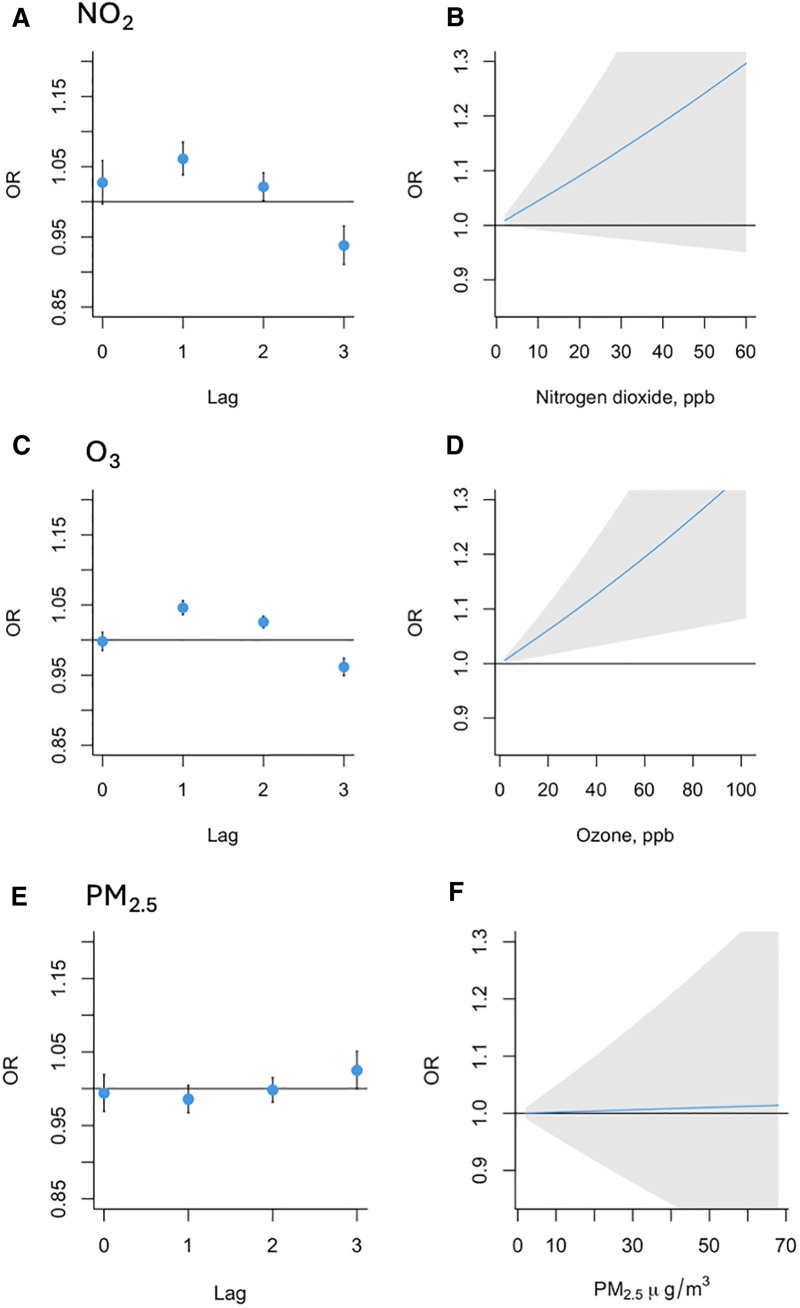
Estimated ORs of migraine onset on days 0–3 after air pollutant exposures, and overall estimated ORs for the 4-day lag period. The specific lag estimated ORs of the association between a relatively higher (A) NO_2_ estimate by 10 ppb NO_2_, (C) O_3_ estimate by 10 ppb, and (E) PM_2.5_ estimate by 10 µg/m^3^, and new migraine attack, on the day of and 3 days after exposure. The overall estimated OR of the association between relatively higher (B) NO_2_ estimate by 10 ppb, (C) O_3_ estimate by 10 ppb, (F) PM_2.5_ estimate by 10 µg/m^3^ and migraine onset over 0-3 days.

Figure 1 presents the associations between NO_2_, O_3_, or PM_2.5_ exposures and migraine attack onset. (A) The first and second days after a day with relatively higher NO_2_ by 10 ppb were associated with 6% and 2% higher odds of new migraine attacks. The 1-day lagged estimate was statistically significant (OR 1.06, 95% CI: 1.03, 1.08). The point estimates for the association on the day of the exposure and two days after were above the null value of 1, with confidence intervals including the null value (OR 1.02, 95% CI: 1.00, 1.06; OR 1.02, 95% CI: 1.00, 1.04). The estimate for the third day after a relatively higher NO_2_ exposure was below the null value and statistically significant (OR 0.93, 95% CI: 0.91, 0.97), suggesting an inverse relation between higher NO_2_ and new migraine events 3 days later. (B) The net association over lags 0–3 was positive, with higher cumulative odds of new migraine onset over 0–3 days after a day with higher NO_2_. The confidence range includes the null value. (C) The first and second days after a day with relatively higher O_3_ by 10 ppb were associated with 5% and 3% higher odds of new migraine attacks. These estimates were statistically significant. (OR 1.05, 95% CI: 1.04, 1.06; OR 1.03, 95% CI: 1.02, 1.03). The point estimate for the association on the day of the exposure was at the null value (OR 1.00, 95% CI: 1.00, 1.01). The estimate for the third day after a relatively higher O_3_ exposure was below the null value and statistically significant. (OR 0.96, 95% CI: 0.95, 0.97), suggesting an inverse relation between higher daily O_3_ and new migraine events three days later. (D) The net association over lags 0–3 was positive, with higher odds of new migraine onset over 0–3 days after a day with higher O_3_. The confidence range does not include the null value. (E) All estimated associations with new migraine attacks on days 0–3 after a relatively higher PM_2.5_ exposure by 10 µg/m^3^ were close to 1 and included the null value in their confidence intervals (OR 0.99, 95% CI: 0.99, 1.00; OR 0.99, 95% CI: 0.98, 1.02; OR 1.00, 95% CI: 0.98, 1.01; OR, 95% CI: 1.02 [0.99, 1.05]). These results suggest that PM_2.5_ was not statistically significantly associated with new onset of migraine attacks. (F) The net association over lags 0–3 closely followed the null value 1.0, with no statistically significant change in new attack onsets over 0–3 days after a day with higher PM_2.5_.

**Figure 2.**
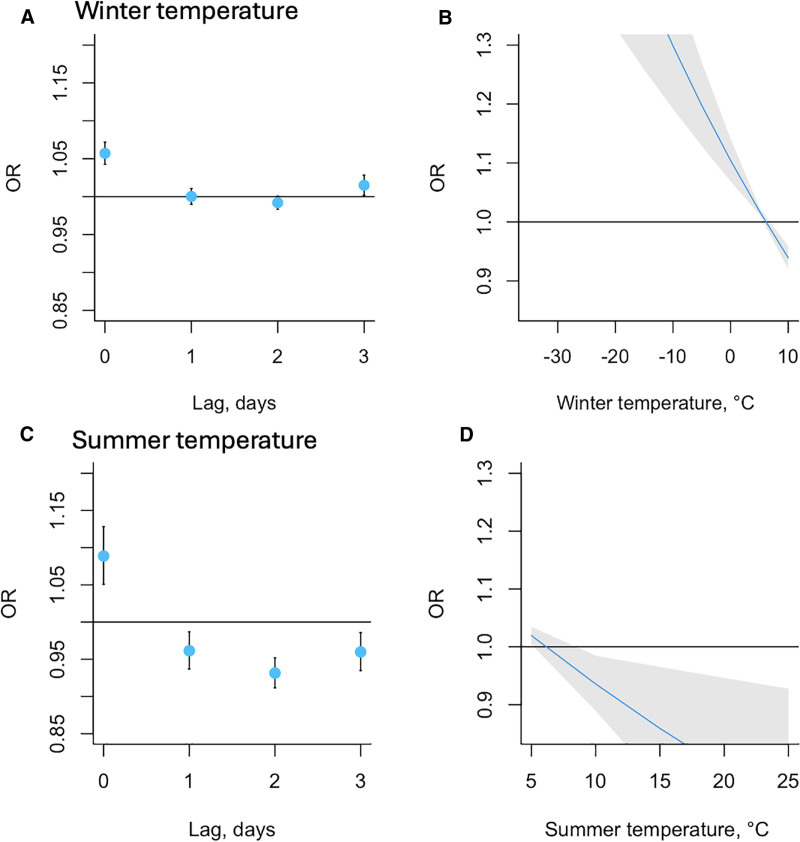
Winter cold and summer heat. The estimated OR of the association between (A) a relatively lower winter temperature by 10°C (C) higher summer temperature by 10°C and new attack onset, on the day of and three days after exposure. The overall estimated OR of the association between (B) a relatively lower winter temperature by 10°C on day 0 (D) a relatively higher summer temperature by 10°C and new attack onset over 0–3 days.

Figure 2 presents the relation between morning (7 am) temperature and migraine attack onset, analyzed separately for winter cold and summer heat. A, Winter days with a relatively colder temperature by 10°C were associated with a 6% higher odds of new migraine records. This estimate was statistically significant OR 1.06, 95% CI (1.04, 1.07), Point estimates for the 1–3 days after the exposure were at or close to the null value of 1: OR 1.00, 95 % CI (0.99, 1.01); OR 0.99, 95 % CI (0.98, 1.00); OR 1.02, 95 % CI (1.00, 1.03). B, Centered on the annual average of 6.1°C, the net association between temperature drop by 10°C and migraine onset over lags 0–3 was positive. New migraine onset was more likely after colder winter days. C, An increase of 10° in summer temperature was associated with an 9% higher odds of new onset migraine records on that same day (OR 1.09, 95% CI: 1.05, 1.13). The odds associated with increased temperature at lags 1–3 were all below the null and were also statistically significant (1 day lag: OR 0.96, 95% CI: 0.93, 0.99; 2 day lag: OR, 95% CI: 0.93 [0.91, 0.95]; 3 day lag: OR 0.96, 95% CI: 0.93, 0.99), suggesting an inverse relation between lagged higher daily temperatures and new migraine events compared with same-day. D, The net association between day 0 higher temperature and migraine onset over lags 0–3, centered on the annual average of 6.1°C, was negative.

Given that O_3_ can act as a mediator between high temperatures and negative health impacts,^39,40^ we ran a post hoc analysis of summer temperatures excluding O_3_ from the model. Results were virtually identical to those with the full model (see Supplementary Content; https://links.lww.com/PRSGO/E873).

**Figure 3.**
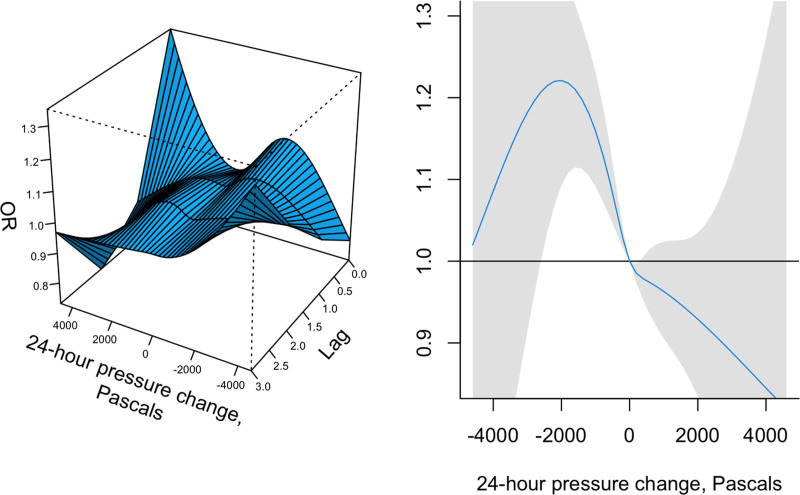
(A) The nonlinear exposure-lag response in estimated OR of association between 24-hour changes in barometric pressure and new migraine onset on the day of, and three days following, 24-hour changes in barometric pressure. (B) The overall estimated OR of the association between changes in barometric pressure and new migraine onset over 0–3 days.

Figure 3 presents the relation between 24-hour change in barometric pressure and risk of migraine onset. A, Days with a 2,000 or more Pascals increase in barometric pressure were more likely to be days on which new migraine attacks were reported. Days with drops of −1,000 to −2,000 in barometric pressure were also more likely to be days on which new migraine attacks were reported. There was limited evidence of lagged associations of migraine with barometric pressure change. B, Centered on 0, or no change over 24 hours, the net association over lags 0–3 peaked around a −2,000 Pa drop in pressure, and dropped with positive changes in pressure.

## Discussion

Our primary objective was to better understand how migraine attack onset may be related to short-term weather and air pollution exposures. We also aimed to explore the use of smartphone app data in a population health setting. In this group of smartphone app users located across Ontario, the net associations across cumulative 0–3 day lags suggested positive associations with higher NO_2_, higher O_3_, colder winter temperatures, and drops in barometric pressure. Cumulative 0–3 day associations with higher summer temperatures were negative, and with PM_2.5_ were null.

For specific lags, when controlling for each other and PM_2.5_, relatively higher NO_2_ and O_3_ were also associated with migraine attack onset. Colder winter temperatures were associated with increased likelihood of migraine attack onset, while days with relatively higher summer temperatures were also associated with increased migraine attack onsets. Positive and negative changes in barometric pressure were associated with migraine attack onset. The magnitudes of specific lag associations ranged from OR 1.09 to 1.03, which is similar in strength to many health outcomes related to air pollution exposure.^17^ Notably, the cumulative lag for higher summer temperatures was negative, whereas the same-day association was positive. This was a mathematical result of negative associations over the following three days. Speculatively, this may be due to event anticipation, a similar process to that known as “harvesting” for terminal outcomes. Harvesting or event anticipation may occur when a stressor affects only susceptible individuals, whose events are merely brought forward.^41^ In this case, a rebound period below the baseline negates the initial increase due to depletion of susceptible individuals.^42^ Given the negative overall 4-day association with warmer summer temperatures after the same-day positive association, warm summer temperatures may hypothetically be bringing migraine events forward rather than triggering them.

There is molecular research to support the physiologic basis of meteorologic triggers for migraine. Primary sensory neurons express transient receptor potential channels, which specialize in sensing pain due to hot or cold temperatures.^43,44^ Activated transient receptor potential sensors trigger the release of CGRP.^44^ When released from the trigeminal nerves, CGRP is part of the pathway to migraine^43^ and a target for migraine-preventive drugs.^45^ This forms the suspected pathway from exposure to heat or cold to induction of migraine via the release of CGRP and resulting neurogenic inflammation. The molecular pathway from NO_2_ exposure to migraine (or migraine-like) behaviors and physiology has recently been explored in rats and humans. Ye et al^15,16^ observed migraine-like behaviors in rats exposed to NO_2_. After NO_2_ treatment, purified rat trigeminal neuron cells expressed more CGRP.^16^ Further experiments demonstrated that the purified trigeminal neuron cell line expressed more CGRP because high insulin-like growth factor 1 (IGF-1) was suppressed.^16^ Genetically high IGF-1 is associated with lower risk of migraine in human studies.^46^ In rat studies, nasally delivered IGF-1 reduces oxidative stress, which in turn inhibits cortical spreading depression.^47–49^ Similarly, participants exposed to relatively cleaner air and then traffic pollution in a crossover study showed an increase in inflammation-related microRNAs.^50^ Overall, the above studies demonstrate that the pathway from air pollution to migraine is likely mediated by the body’s pro-inflammatory reaction to oxidative stress.

This work presents innovative comparisons of the temporal relation between air pollution and weather with migraine. In this study, the lagged relation between weather and pollution differed, with temperature and barometric pressure associated with same-day effects, whereas NO_2_ and O_3_ were associated with the highest odds of migraine attacks at 1- and 2-day lags. The physiology underlying these differences is not known, but for temperature, this may speculatively relate to hypothesized temperature-migraine associations mediated by nerve sensing,^9–12^ which might occur more rapidly compared with putative pathways triggered by air pollution via oxidative stress and inflammation.^7,16^

To our knowledge, this is the first migraine study to model more than two pollutants at once. The 10 km2 small areas were more local compared with previous work—which employed city-wide averages—and likely improved the accuracy of exposure estimates. Additionally, study locations across much of Ontario lent a wide variety of urban and rural locations. Moreover, the fact that participants’ attacks were linked to their g locations at attack onset is likely to further improve air pollution exposure estimates compared with home addresses linked to city-wide exposures. Migraine Buddy users generally record their attacks within 24 hours of the attack onset,^20^ and 4,429 (59.7%) of participants recorded events in more than one 10 km2 location, suggesting that our location data may be more current than a home address. This study also demonstrates the application of a 69,808-event voluntary smartphone app dataset in epidemiologic research, which allowed for individual-level analyses.

When considering multipollutant models, it is important to note that air pollution occurs in mixtures, and it may be unrealistic to conceptualize individual pollutants and meteorological exposures separately.^17,51^ For example, modeling a 10 ppb increase in NO_2_ without a corresponding increase in PM_2.5_ (but conversely holding PM_2.5_ at its average level) may represent an unrealistic scenario. Additionally, some pollutants may be on the pathway between other pollutants and our outcome. For example, higher levels of O_3_ may mediate the health effects of higher temperatures.^17^ As a result, in multipollutant models, estimates for pollutants more distal to migraine might be over-adjusted. Modeling multiple individual pollutants simultaneously may also amplify attenuation of effect estimates based in measurement error.^52^ Finally, health impacts may be more strongly associated with mixtures, leading to underestimation of impacts when controlling for co-pollutants as opposed to analyzing impacts of combinations.^51,53^ This is illustrated in the migraine context by Dong et al,^54^ who described stronger associations of incident migraine diagnosis with a combined-pollutant air quality mixture measure than for individual pollutants. Exploring methods to simultaneously model multiple pollutants and their interactions with each other is an important area for future work, assessing how air pollution may impact migraine.

Another strength of this study is the use of the case-time series, which allowed comparison of participants to themselves within a given month of a year, thereby accounting for the fact that individuals’ engagement with the app may change over time. Accounting for user drop-out within the analysis is important because app users may stop recording their attacks at any time, which is a weakness in this data source. This work is based on the assumption that individuals were likely to record attacks consistently within a given month, and allows for joining and attrition between months. More generally, the study design offers strong control for both individual-level differences and temporal trends.

A common drawback of smartphone app data is a lack of demographic, social, and economic information. In this study, both gender and age had substantial missing data: 26.3% and 67.1%, respectively. Another mobile health app reported ~30% missing sociodemographic data.^55^ Stable individual-level characteristics are controlled by design in the case-time series. However, factors such as age, gender, education, and other socioeconomic factors are important reflections of the sample population, hold future interest as effect modifiers. Future studies should address the potential effect modification between pollutants and migraines across these measures. The fact that most participants were women and the lack of socioeconomic status information impacts generalizability to other genders and individuals who do not use smartphones.

All of the environmental estimates in this study were measured once-daily.^21,22,56,57^ Since O_3_ is highly variable throughout the day and peaks during sunlight hours, the best-practice in O_3_ estimation is the 8-hour maximum.^58,59^ Therefore, our single-point estimates may not accurately assess the exposure, leading to measurement error and potential exposure misclassification bias. This would be expected to decrease the likelihood of observing associations with migraine onset. That our results nonetheless point to a statistically significant association suggests that these results are robust to some mismeasurement of O_3_. Future studies should consider using more precise exposure estimates such as the 8-hour O_3_ maximum. Another way to estimate health impacts of O_3_ and NO_2_ is through combined oxidant capacity,^60^ which may be used in future studies of air pollution and migraine.

Our results build upon previous single- and two-pollutant studies. Earlier studies in this area suggested that associations with NO_2_ would be stable when controlling for other individual pollutants.^1^ We present a novel report of statistically significant associations for NO_2_ and O_3_ in a multipollutant model. Previous work suggested that statistically significant associations with PM_2.5_ may be partially confounded by uncontrolled confounding by colinear NO_2_ pollution.^1,61,62^ These results are borne out by our finding that PM_2.5_ was not statistically significantly associated with migraine onset after controlling for NO_2_ and O_3_, unlike in a single-pollutant model (See Supplementary Content; https://links.lww.com/PRSGO/E873).

In the days following, estimated positive associations with NO_2_, O_3_, and temperature, associations appear inverted. Results from our simulation study (see Supplementary Content; https://links.lww.com/PRSGO/E873) suggest that this inversion is not exclusively due to the 2-day risk-free period after a migraine attack. There are several other possible contributors to this pattern. One is that the environmental triggers may have aggravated migraine attacks that were already in the prodrome phase. It is also possible that on the days after the exposures, most of the participants who were sensitive to the given exposure were continuing to experience an attack. Alternatively, given that the previous day’s exposure often influences the next, participants may have adjusted to the exposure over time, for example, if hot summer weather continued for three days. Future work may differentiate these potential causes.

Most previous work has used emergency room and clinic visits. Both emergency room and smartphone data have strengths and weaknesses and give different insights. Migraine diagnoses by doctors in the emergency room are more likely than self-reports to meet the ICD-10 criteria for migraine. They are also more likely to include severe or intractable symptoms. Migraine Buddy provides symptom checklists, but not explicit diagnostic criteria within the user interface. Our study assumes that most of the Migraine Buddy users who report migraine events are, in fact, experiencing migraine. Previous publications have reported excellent agreement between self-reported migraine and meeting ICHD-3 among female cohorts, ranging from 82%–87%.^63,64^ In a survey related to ER use for migraine among Migraine Buddy users, there was 94% agreement between self-reported migraine and either meeting ICHD-3 or having been diagnosed by a doctor.^20^ An advantage of the app data is clearer records of onset time, compared with ER visits, where a large portion of patients arrive at the ER days or up to a week after onset.^18–20^ Despite Migraine Buddy users recording most attacks linked to ER visits and across a range of severity,^20^ we expect that the majority of attacks recorded in a smartphone app would be significantly less severe in terms of pain level and disability than events that lead to ER visits.

The magnitude of estimated associations here is relatively minor, ranging from 2% to 9% increases in the odds of attack. However, Ontario has a population of over 16 million,^65^ of which an estimated 8.3% live with migraine.^66^ Therefore, if these results were to be generalizable, even a 2% increase in the likelihood of onset would represent a large number of migraine events in this jurisdiction that could be potentially forewarned by predicted environmental conditions.

The most effective way to reduce air pollution exposures is through policy,^67^ such as industrial and vehicle emission control measures.^68^ However, in the same way that migraine patients already take preventive treatment for menstrual migraine or avoid dehydration day to day, clinicians may advise patients who are sensitive to air pollution to avoid or plan for times and places with high air pollution. Based on these results, the Migraine Buddy team may integrate air pollution sensitivity estimates into the Migraine Buddy app.

Multipollutant studies in jurisdictions or time periods that continue to experience relatively higher particulate matter—such as from wildfire in Ontario or in emerging economies—may give insight into discrepancies between previous studies and our results. Other areas of future study include integrating pollen and noise as exposures, and neighborhood greenness and socioeconomic factors as potential effect modifiers.

## CONCLUSIONS

We provide evidence from multiexposure modeling with a dataset including 69,808 health events that migraine attacks are more likely to begin after exposure to relatively higher NO_2_ or O_3_ pollution, relatively colder winter temperatures or warmer summer temperatures, and changes in barometric pressure. This work also demonstrates innovative integration of local environmental exposures, smartphone app data for a transient neurologic outcome, and the case-time series design for environmental epidemiology.

## ACKNOWLEDGMENTS

This project was carried out in collaboration with the Migraine Buddy research team at Aptar Inc. Our collaborators at Migraine Buddy did not review or comment on the results or manuscript. The authors are grateful for support from Nadia Muhe and Zeynep Cevik, Statistical Support Specialists, and Cole White, GIS Analyst, at the University of Toronto Map and Data Library. Thanks to the SPHERE lab at Boston University for thoughtful feedback and to Ioana Nicolau at the University of Calgary for critical review of the manuscript. We are grateful to the Open Data Access team and Environment and Climate Change Canada for supplying at-cost daily weather and pollution surfaces.

C.L. received the University of Toronto Slamen Fast Grant 2023 for research and the Women’s College Hospital practice grant 2023 for research. She has received consulting fees from Abbvie, Pfizer, Teva, and Lundbeck, and payment or honoraria from Pfizer, Lundbeck, and Abbvie. She is a board member of the American Headache Society and the Canadian Headache Society since 2022. She is the chair of the American Migraine Foundation. All these positions are unpaid. The other authors have no conflicts to report.

## Supplementary Material
