# Supplementary material for: Weather, air pollution, and migraine: A case-time series analysis examining environmental exposures and transient health outcomes recorded via smartphone application
Source: Environ Epidemiol. 2026 May 8;10(3):e475. doi: 10.1097/EE9.0000000000000475 (PMC13155513; doi:10.1097/EE9.0000000000000475)
Supplement: Supplementary file 1 [file ee9-10-e475-s001.pdf]

Supplemental content

Temperature and humidity

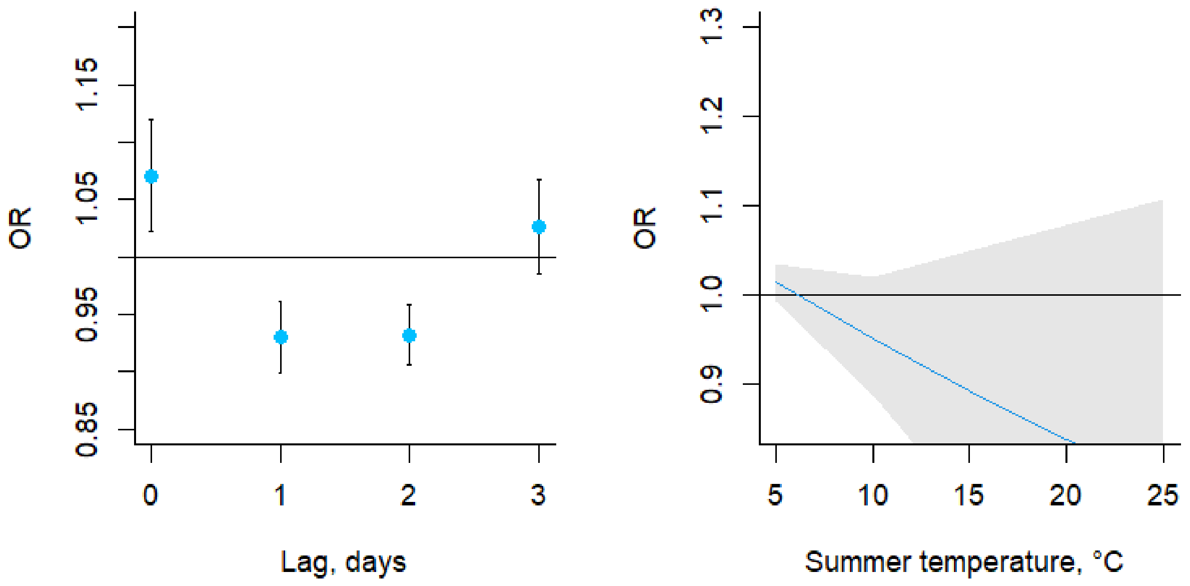

Supplementary Figure 1: Summer heat controlling for humidity. a) The estimated odds ratio of the association between a relatively higher temperature by 10 °C and new migraine onset, on the day of and three days following exposure. b) The overall estimated odds ratio of the association between a relatively higher temperature by 10 °C on day 0 and new migraine onset over 0-3 days.

In our main analysis, humidity was excluded from the model during initial steps of model building due to high redundancy with temperature and the fact that inclusion of humidity greatly increased the AIC (decreased parsimony). We conducted a post-hoc analysis of temperature effects including humidity in the model. As seen in

Supplementary Figure 1a, in comparison to Figure 2c, including humidity somewhat attenuated the association at Lag 0, while overall estimates remained quite similar. Point estimates for lags 0-3 were 1.07 (95 % CI 1.02, 1.12), 0.93 (95 % CI 0.89, 0.96), 0.93 (95 % CI 0.91, 0.96), and 1.03 (95 % CI 0.99, 1.07) respectively. b) The cumulative lag-response also remained similar to the main analysis.

Temperature and Ozone

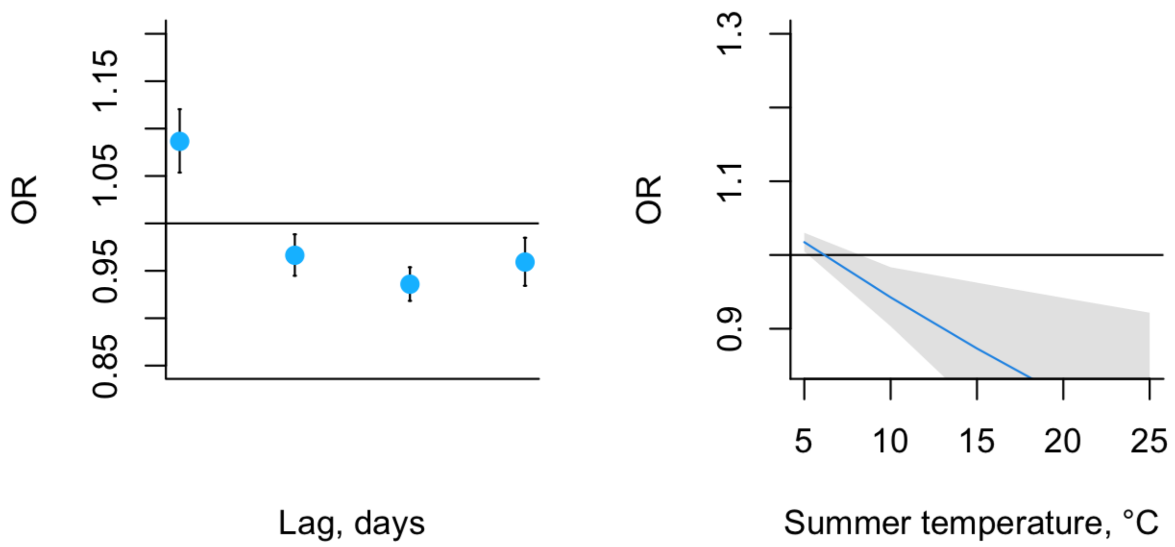

Supplementary Figure 2: Summer heat in a model excluding O_3. a) The estimated odds ratio of the association between a relatively higher temperature by 10 °C and new migraine onset, on the day of and three days following exposure. b) The overall estimated odds ratio of the association between a relatively higher temperature by 10 °C on day 0 and new migraine onset over 0-3 days.

Supplementary Figure 2 presents the relationship between morning (8 AM) summer temperature and migraine onset. Estimates are centered around the mean annual temperature of 6 °C, presumed to be the minimum risk temperature. a) Similarly to when O_3 was included in the model, an increase in 10 °C in temperature was associated with 9 % higher odds of new onset migraine records on that same day (OR 1.09, 95 % CI (1.05, 1.12). The odds associated with increased temperature on up to three days preceding the migraine event were all below the null and were also statistically significant (1 day lag: OR 0.97, 95 % CI (0.94, 0.99); 2 day lag: OR, 95 % CI 0.93 (0.91, 0.95); 3 day lag: OR 0.96, 95 % CI (0.93, 0.98)), suggesting. a) Also in agreement with the full model, the net association between day 0 higher temperature and migraine onset over lags 0-3 was negative. These results suggest that the associations between air pollution and migraine are not mediated by O_3, which is also in agreement with the fact that temperature associations appear to be same-day, whereas estimated associations of migraine onset with O_3 were positive on the first and second day following exposure.

Temporary post-outcome censoring

Methods

Due to the clinical nature of migraine,[12–14] participants were not deemed to be at risk of a new migraine attack for the 2 days following a recorded attack. This period without risk of the outcome partly violates the key assumption of outcome-independent follow-up.[35] Importantly, this 2-day risk-free period left individuals at

risk of migraine during the referent days, which were the same weekdays in the same month (for example, the other Mondays in June). However, the 2-day recovery period could have potential to introduce bias to our analysis. Therefore, we examined potential bias introduced by this risk-free period through a simulation study. Our simulation study built on the simulations presented in the 2021 article *Case Time Series Design*,[18] by modifying Scenario 12: Outcome-dependent follow-up. The simulation Scenario 12 modeled a sample exposure and outcome in 500 subjects over 1 year.[18] We modified our scenario to include a 0-3 day exposure-response lag, and a 2-day risk-free period following recorded attacks.

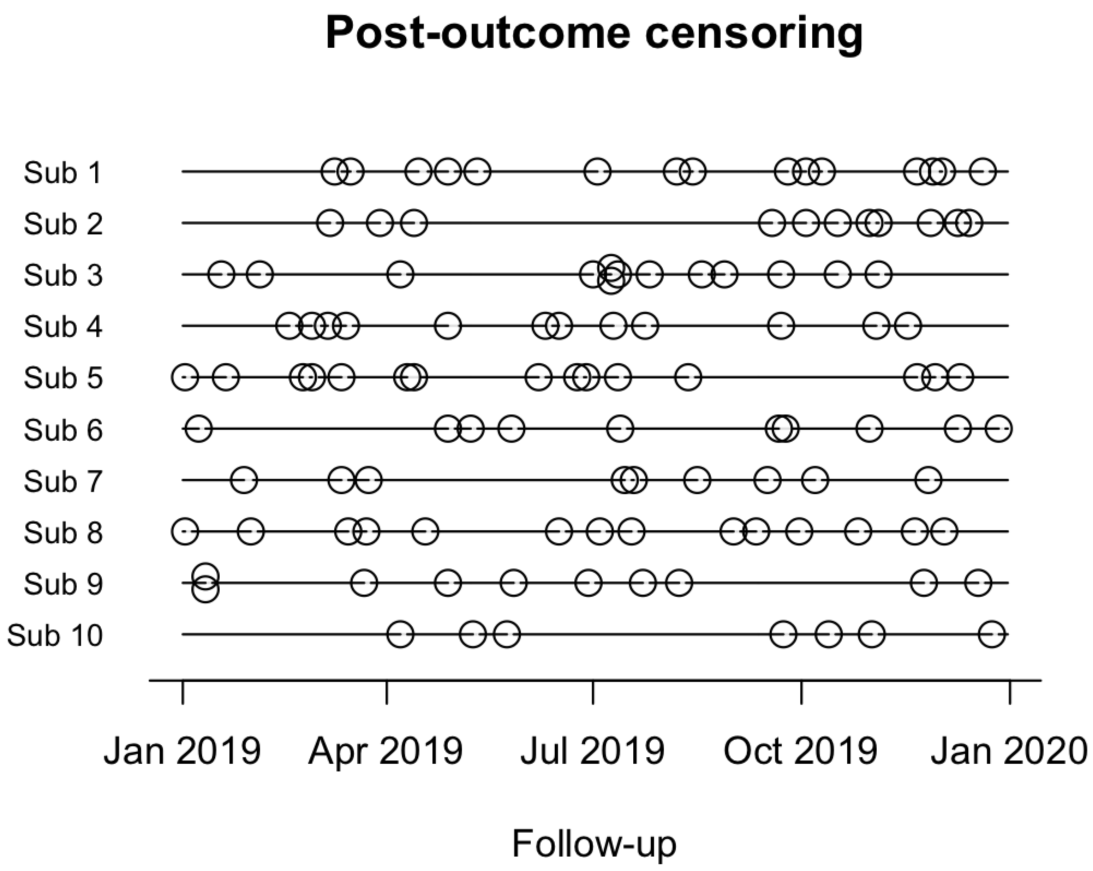

Supplementary Figure 3: This figure illustrates the simulated data for 10 subjects with 5-10 events over a calendar year. Migraine onset events are shown as circles; the censored periods are shown as gaps in the timeline following each event.

Supplementary Figure 3 shows ten simulated participant timelines with events (circles) and post-outcome censoring (gaps in lines). We ran a simulation study to quantify the magnitude and direction of bias induced by the two-day risk-free period. The results suggest a relative bias of 0.06 %. The predicted coverage (proportion of times the confidence interval overlaps the true value)[49] was 93.8 %. The estimated

root mean square error was 16.1 %. Taken together, these results suggest that post-attack censoring introduces minimal bias to our analysis.

Single vs multi-pollutant models

Supplementary Table 1 : Comparison of single- and multi-pollutant models.

Pollutant	Lag, days	**Single-pollutant models, OR (95 % CI)**	**Multi-pollutant model, OR (95 % CI)**
NO_2	Lag 0	OR 1.03 (1.01, 1.04)	OR 1.02 (1.00, 1.06)
	Lag 1	OR 1.01 (1.00, 1.03)	OR 1.06 (1.03, 1.08)
	Lag 2	OR 1.00 (0.99, 1.01)	OR 1.02 (1.00, 1.04)
	Lag 3	OR 0.98 (0.97, 1.00)	OR 0.93 (0.91, 0.97)
O_3	Lag 0	OR 0.99 (0.98, 1.01)	OR 1.00 (1.00, 1.01)
	Lag 1	OR 1.03 (1.02, 1.04)	OR 1.05 (1.04, 1.06)
	Lag 2	OR 1.02 (1.01, 1.03)	OR 1.03 (1.02, 1.03)
	Lag 3	OR 0.98 (0.97, 0.99)	OR 0.96 (0.95, 0.97)
$PM_{2.5}$	Lag 0	OR 1.02 (1.01, 1.04)	OR 0.99 (0.99, 1.00)
	Lag 1	OR 1.02 (1.01, 1.03)	OR 0.99 (0.98, 1.02)
	Lag 2	OR 1.01 (1.00, 1.02)	OR 1.00 (0.98, 1.01)
	Lag 3	OR 0.98 (0.97, 1.00)	OR 1.02 (0.99, 1.05))

Supplementary Table 1 shows estimated associations of migraine onset with single-pollutant and multi-pollutant models. Estimates for NO_2 and O_3 remain relatively stable, whereas most estimates for $PM_{2.5}$ are attenuated in the model that also includes NO_2 and O_3.
